# Nanoporous silicon fiber networks in a composite anode for all-solid-state batteries with superior cycling performance

**DOI:** 10.1038/s41598-023-44070-1

**Published:** 2023-10-10

**Authors:** Mari Yamamoto, Mika Takatsu, Ryota Okuno, Atsutaka Kato, Masanari Takahashi

**Affiliations:** 1 Osaka Research Institute of Industrial Science and Technology, Morinomiya Center, 1-6-50, Morinomiya, Joto-ku, Osaka-City, Osaka 536-8553 Japan; 2 Graduate School of Materials Science, Nara Institute of Science and Technology, 8916-5 Takayama-cho, Ikoma, Nara 630-0192 Japan

**Keywords:** Batteries, Nanowires

## Abstract

All-solid-state batteries comprising Si anodes are promising materials for energy storage in electronic vehicles because their energy density is approximately 1.7 times higher than that of graphite anodes. However, Si undergoes severe volume changes during cycling, resulting in the loss of electronic and ionic conduction pathways and rapid capacity fading. To address this challenge, we developed composite anodes with a nanoporous Si fiber network structure in sulfide-based solid electrolytes (SEs) and conductive additives. Nanoporous Si fibers were fabricated by electrospinning, followed by magnesiothermic reduction. The total pore volume of the fibers allowed pore shrinkage to compensate for the volumetric expansion of Li_12_Si_7_, thereby suppressing outward expansion and preserving the Si-SE (or conductive additive) interface. The network structure of the lithiated Si fibers compensates for electronic and ionic conduction pathways even to the partially delaminated areas, leading to increased Si utilization. The anodes exhibited superior performance, achieving an initial Coulombic efficiency of 71%, a reversible capacity of 1474 mAh g^−1^, and capacity retention of 85% after 40 cycles with an industrially acceptable areal capacity of 1.3 mAh cm^−2^. The proposed approach can reduce the constraint pressure during charging/discharging and may have practical applications in large-area all-solid-state batteries.

## Introduction

The invention of Li-ion batteries is a key factor in the development of portable electronic devices and electric vehicles. Further increases in demand have led to the need for higher energy densities^1^. Si is widely considered the most promising anode material for increasing energy densities owing to its low potential (< 0.35 V vs. Li^+^/Li) and large theoretical capacity (3572 mAh g^−1^ for Li_15_Si_4_ vs. 372 mAh g^−1^ for conventional graphite)^2,3^. Given the abundance of Si in the crust of the Earth, further increases in the demand for this element can be satisfied. However, Si undergoes significant volume changes during lithiation and delithiation, leading to Si particle pulverization, continuous solid electrolyte interface (SEI) growth, and, in turn, rapid capacity reduction. Current efforts to overcome these challenges and mitigate capacity fading include the use of nanoparticles^4,5^, nanowires^6–8^, nanotubes^9^, nanosheets^10^, and hollow particles^11^. These strategies can improve the cyclability of the resulting materials; however, their practical application is limited by the low initial Coulombic efficiency (ICE). Li^+^ is consumed by the decomposition of the liquid electrolyte on Si surfaces with large specific surface areas, causing significant SEI growth^12^. Sulfide-based solid electrolytes (SEs) may overcome this limitation by minimizing SEI growth owing to their ability to form stable and passivating SEIs^13,14^. Moreover, non-flowable SEs are unlikely to seep into the cracks of SEIs on Si surfaces and form new SEIs. Despite these promising advantages, the applications of Si anodes in all-solid-state batteries using SEs are rarely studied^15^.

Thin Si films (thickness, < 300 nm)^16^ exhibit high capacity and stable cyclability. Porous^17^ and columnar^18^ Si films with industrially relevant areal loadings display superior cycling stability, indicating that the spaces in the porous and columnar structures are effective for stress relaxation. Such results also show that electrons and Li^+^ can be conducted through Si films without SEs and conductive additives. The diffusion coefficient of Li within lithiated Si is approximately ~ 10^−11^ cm^2^ s^−1^^19^, which is similar to that of the commonly used cathode material, i.e. LiCoO_2_ (10^−12^–10^−11^ cm^2^ s^−1^)^20^. During lithium insertion, the electronic conductivity of Si (6.7 × 10^−4^ S cm^−1^^21^) increases by approximately 3.5 orders of magnitude^22^ to a value similar to that of the typical cathode materials, LiNi_1/3_Mn_1/3_Co_1/3_O_2_ (10^−6^–10^−3^ S cm^−1^)^23^. We previously studied the performance of composite anode sheets fabricated from microsized Si particles, an SE, and a conductive additive in a slurry^24^. This process was cost-effective, vacuum-free, and applicable to large-area continuous mass manufacturing; additionally, it achieved active material loadings per unit area similar to that of commercial LIBs. Vertical cracks, which opened and closed repeatedly, were formed in the electrodes during charging/discharging, leading to effective stress relief. However, reductions in the constraint pressure of the cell during charging/discharging disrupted the interface between Si and the SE or conductive additive, resulting in severe cycling degradation during the first few cycles^25^. Recent studies show that composite anodes with nanoporous Si particles (diameter, ~ 500 nm) exhibit improved cyclability, indicating that pores can act as a buffer space for volume change^26–29^. Therefore, the development of Si composite anodes with well-maintained ionic and electronic conduction pathways is key to their application in all-solid-state batteries.

**Figure 1:**
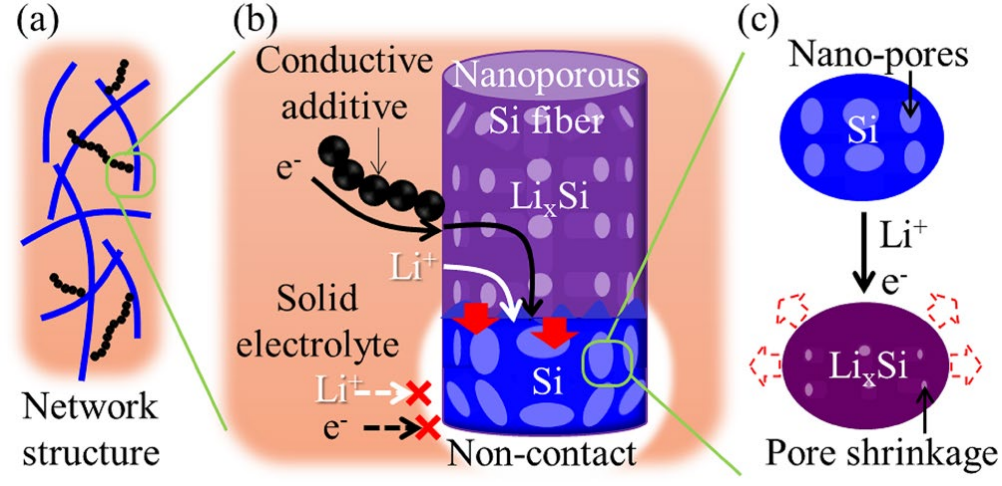
Schematic of the strategy for achieving stable cycling performance. (**a**) Composite anode with a network structure of nanoporous Si fibers. (**b**) Electron and ion conduction occurs through the lithiated Si fibers to increase Si utilization. (**c**) Accommodation of volume expansion by pore shrinkage.

In this study, we describe a new strategy that takes advantage of both porous and fibrous network structures to effectively construct and maintain ionic and electronic pathways throughout a lithiated Si electrode (Fig. 1). The porous structure mitigates the volume change of Si owing to pore shrinkage, resulting in well-maintained interfacial contact between Si and the SE or conductive additives (Fig. 1c). The fibers easily produce a network structure in the composite anode (Fig. 1a). The SE and conductive additive are major factors governing the formation of ionic and electronic conduction paths in the composite anode. The lithiation of Si begins at the contact interface of the SE and conductive additive, enhancing the ionic and electronic conductivities of the resulting Li_x_Si. The partially formed Li_x_Si, can continuously propagate ions and electrons to the partially delaminated area between the Si fibers and SE or conductive additives, leading to increased Si utilization (Fig. 1b). To the best of our knowledge, this is the first study to apply nanoporous Si fibers in all-solid-state batteries.

Nanoporous Si fibers were fabricated by simple electrospinning^30^, followed by magnesiothermic reduction^31,32^. Electrospinning is inexpensive, applicable to continuous production processes, and enables good fiber diameter control. The magnesiothermic reduction of silica to produce porous Si is conducted at a lower temperature (~ 685 °C) than that required for conventional carbothermal reduction (2000 °C)^33^. Therefore, carbothermal reduction should promote the fusion of the precursor fibers into spherical bulk particles; in contrast, magnesiothermic reduction facilitates the inheritance of the micromorphology of the precursors by the products. We successfully prepared nanoporous Si fibers with sufficient pore volumes to accommodate the volume expansion of Li_x_Si during lithiation. A composite anode with a fiber network structure can be easily constructed by mixing the fibers with sulfide-based SEs and conductive additives. An all-solid-state battery prepared with the Si composite as an anode exhibited a relatively high ICE of 71% and stable reversible capacity of 1474 mAh g^−1^ with 85% capacity retention after 40 cycles; additionally, it achieved a capacity of 1038 mAh g^−1^ with a capacity retention of 60% after 200 cycles.

## Results and discussion

### Synthesis and characterization of nanoporous Si fibers

**Figure 2:**
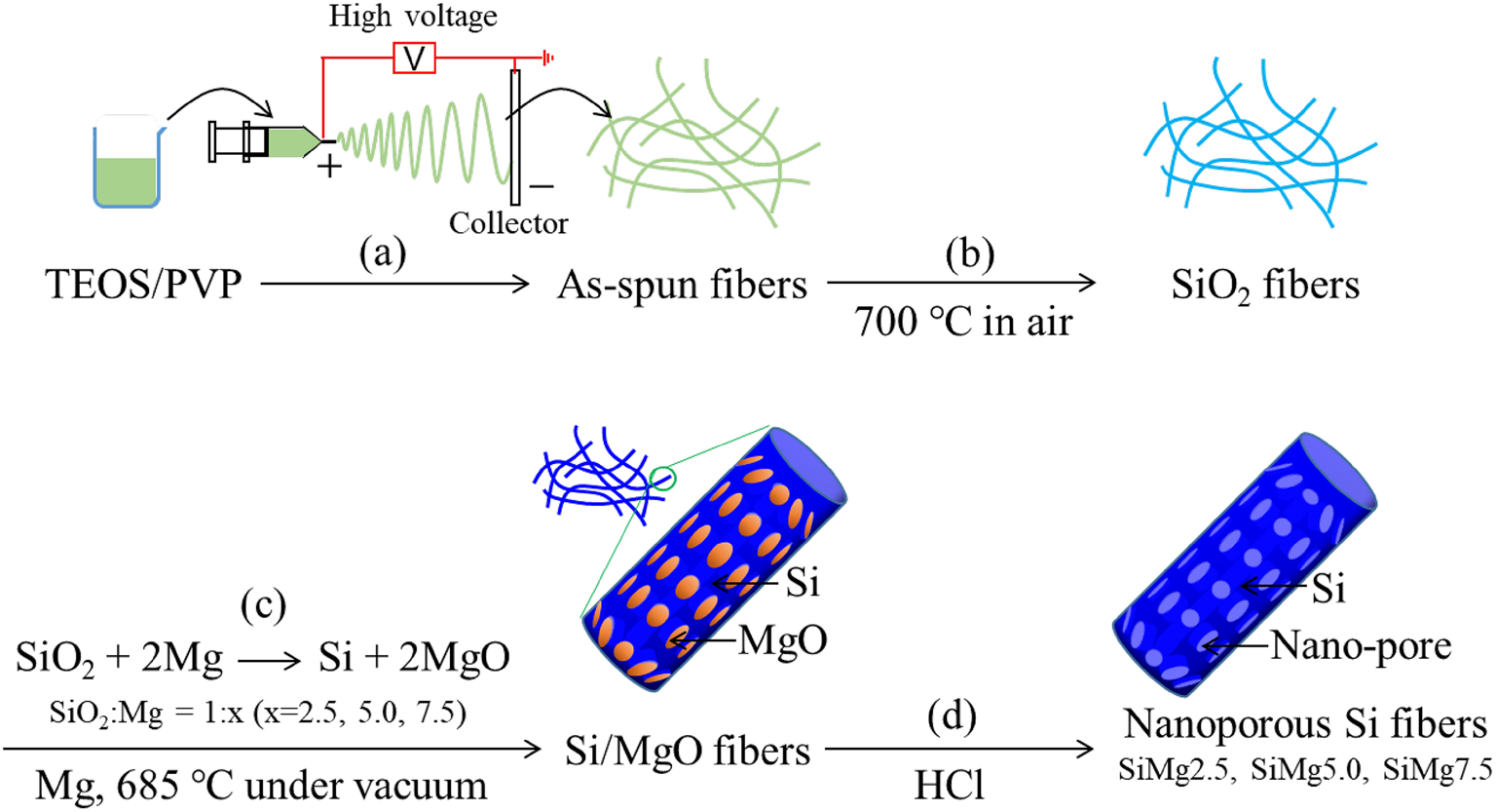
Schematic of the nanoporous Si fiber synthesis process. (**a**) Electrospinning, (**b**) calcination, (**c**) magnesiothermic reduction, and (**d**) HCl etching.

Figure 2 shows a schematic of the synthesis of nanoporous Si fibers by electrospinning, followed by magnesiothermic reduction. The tetraethyl orthosilicate (TEOS)/polyvinylpyrrolidone (PVP) precursor solution was spun to fabricate nonwoven fibers (Fig. 2a), the morphology of which was strongly affected by the relative humidity of the environment during electrospinning^34^. No fibers were formed at humidities above 55%. Interconnected fibers were obtained at approximately 50% humidity (Fig. S1a), whereas independent fibers were obtained at a humidity below 45% (Fig. S1b). These results indicate that the evaporation of water from the electrospinning jet was accelerated when the water vapor concentration in the air was lower than the saturated vapor concentration, resulting in the formation of independent fibers. Fibers prepared at less than 45% humidity were used in subsequent experiments.

The as-spun fibers were peeled from the substrate and calcined in air at 700 °C for 2 h to remove organic components and prepare SiO_2_ fiber sheets (Fig. 2b). The obtained SiO_2_ fiber sheets were then alternately stacked in direct contact with Mg powder and converted to Si/MgO fibers by magnesiothermic reduction (Fig. 2c). We prepared fibers with SiO_2_:Mg molar ratios of 1:x (x = 2.5, 5.0, 7.5) to optimize the reaction conditions and assess the effect of a lack of Mg owing to the escape of Mg vapor under vacuum and/or its reaction with the ceramic boat. Finally, HCl etching was performed to remove MgO and retain the nanoporous Si fibers (Fig. 2d). Nanoporous Si fibers prepared with SiO_2_:Mg molar ratios of 1:x (x = 2.5, 5.0, 7.5) were denoted as SiMgx.

**Figure 3:**
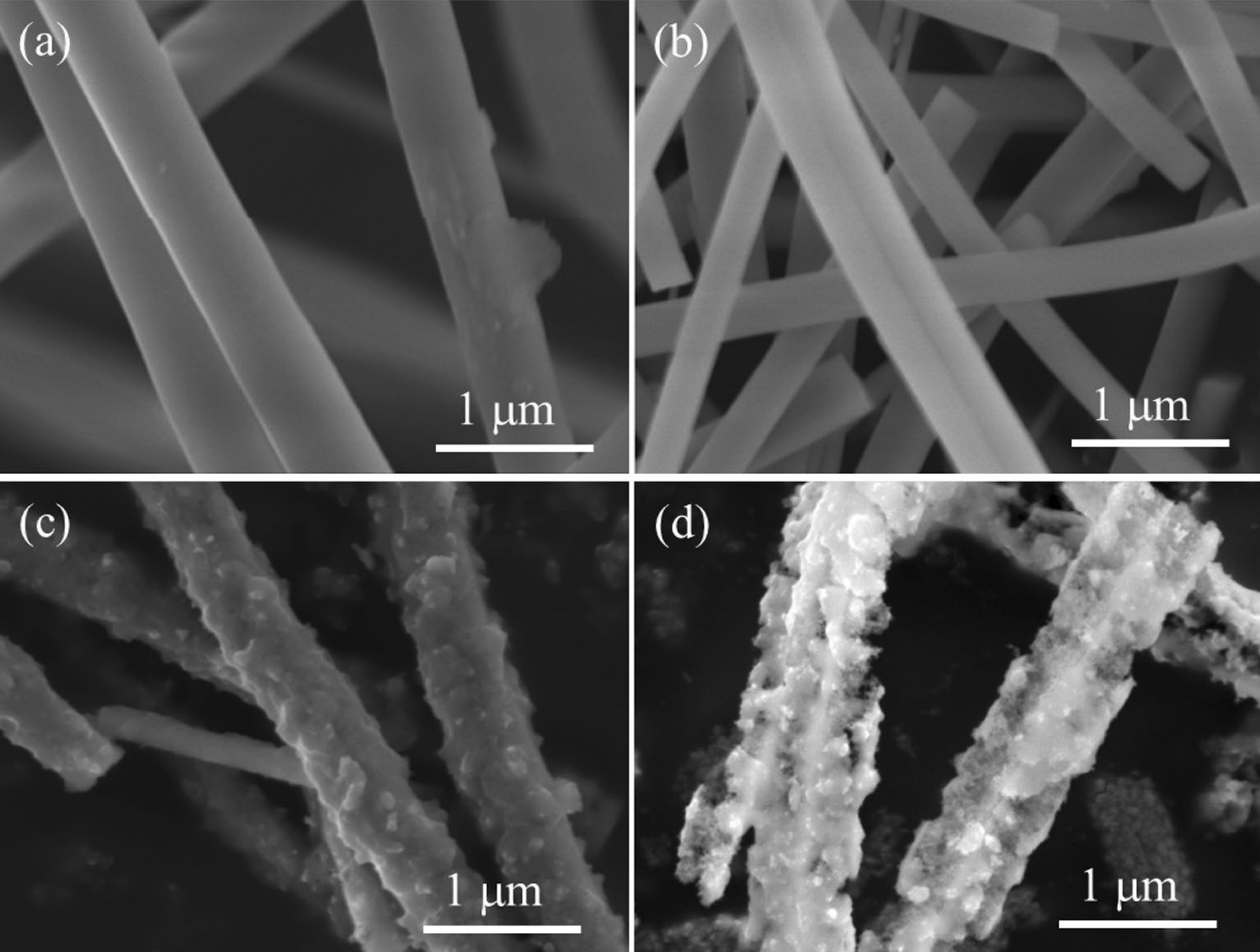
Morphological changes in the nanoporous Si fibers during their preparation. SEM images of the (**a**) as-spun fibers, (**b**) SiO_2_ fibers after calcination in air, (**c**) SiMg5.0 before HCl etching, and (**d**) SiMg5.0.

Figure 3 shows the representative field-emission scanning electron microscopy (FE-SEM) images of the as-spun fibers, SiO_2_ fibers, SiMg5.0 before HCl etching, and SiMg5.0. The as-spun and SiO_2_ fibers contained smooth surfaces and were approximately 1 μm in diameter (Fig. 3a,b). After magnesiothermic reduction, the original morphology and diameter of the fibers were maintained. However, the roughness of the surfaces of the reduced fibers increased with submicrometer precipitates (Fig. 3c). Energy-dispersive X-ray spectroscopy (EDX) mapping showed that Si and Mg were present at the same position in SiMg5.0 before HCl etching (Fig. S2). Quantitative analysis showed that the Si:Mg atomic ratio was approximately 1:2, which is consistent with Eq. 1:

1 $${\text{SiO}}_{{2}} + {\text{ 2Mg }} \to {\text{ Si }} + {\text{ 2MgO}}$$

After HCl etching, a highly porous surface was obtained, and the morphology and diameter of the reduced fibers were maintained (Fig. 3d). The absence of Mg in the EDX spectrum (Fig. S3b) indicates that the fibers were highly porous, which was attributed to the selective etching of the embedded MgO. SiMg7.5 exhibited a similar fiber morphology with highly porous surfaces (Fig. S4a). Conversely, SiMg2.5 had partially smooth surfaces, implying that some of the SiO_2_ precursors remained in the sample owing to insufficient Mg vapor (Fig. S4b).

The porosity of the fibers was characterized using N_2_ gas adsorption measurements (Table S2). SiMg5.0 exhibited a large specific surface area (169 m^2^ g^−1^) and high total pore volume (0.519 cm^3^ g^−1^). The average pore diameter calculated from the specific surface area and total pore volume of the sample was 12.2 nm. The total pore volume (0.519 cm^3^ g^−1^) was consistent with the volume change (0.522 cm^3^ g^−1^) of Si after the formation of Li_12_Si_7_, with a theoretical specific capacity of 1635 mAh g^−1^. Therefore, the outward expansion of the fibers can be suppressed by pore shrinkage at charging capacities of up to 1635 mAh g^−1^. The critical fracture diameter of porous Si particles can reach 1.52 μm^35^. Therefore, nanoporous Si fibers with a diameter of approximately 1 μm can retain their structure without pulverization during charging/discharging. Such a high-porosity fibrous structure would be beneficial for achieving high capacity and long cycle life. The specific surface area (109 m^2^ g^−1^) and porosity (0.266 cm^3^ g^−1^) of SiMg7.5 were lower than those of SiMg5.0. The molar ratio of Mg/SiO_2_ strongly affects the composition^36,37^ and porosity^38^ of the fibers. A high Mg molar ratio resulted in a high concentration of Mg vapor; this generated a local high-temperature environment owing to the rapid exothermic reaction. Therefore, the reduction in fiber porosity was attributed to the accelerated fusion and growth of Si crystals, leading to the absence of pores.

**Figure 4:**
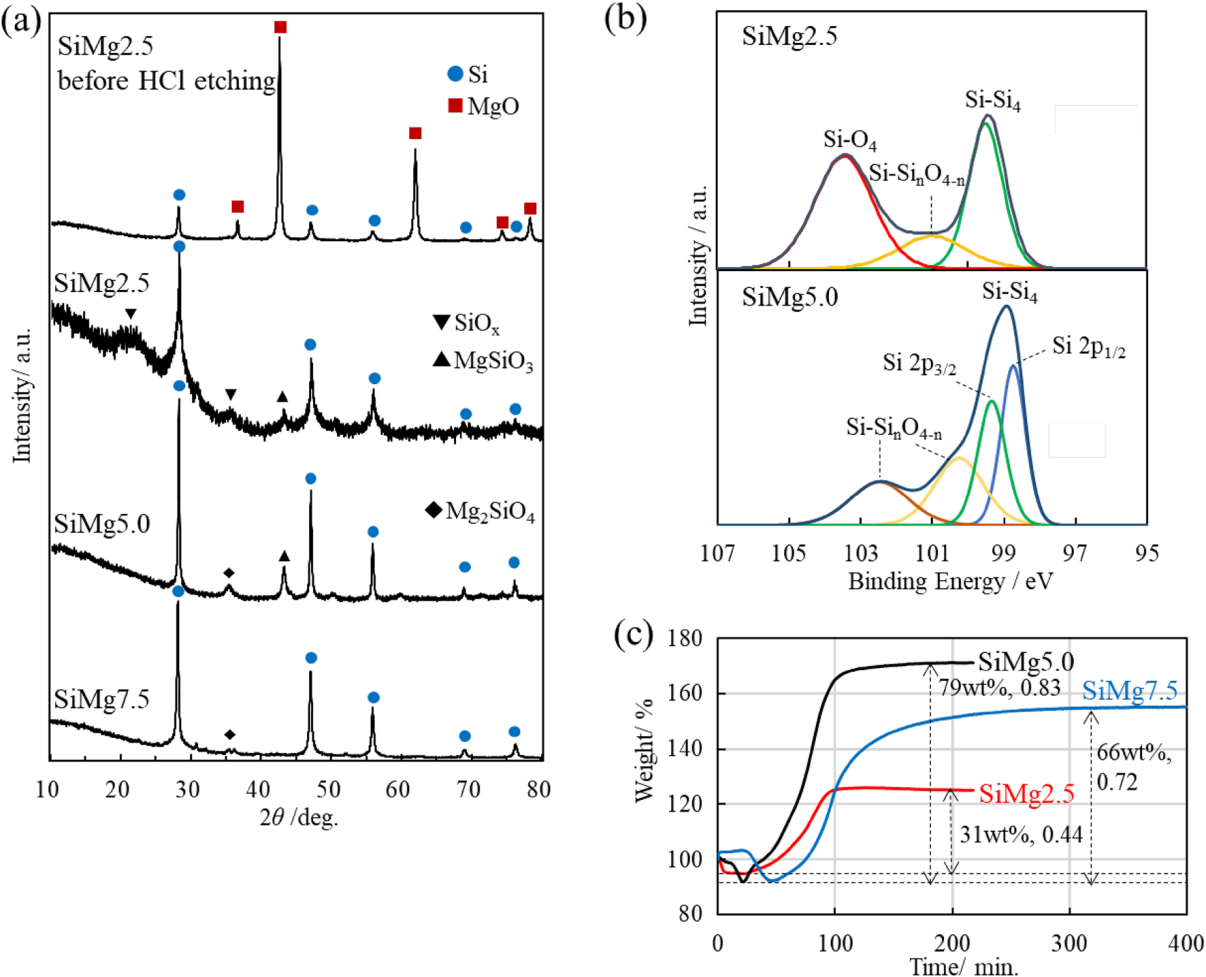
Characterization of nanoporous Si fibers. (**a**) XRD patterns of nanoporous Si fibers in SiMg2.5 before HCl etching and SiMg2.5, SiMg5.0, and SiMg7.5. (**b**) XPS spectra of the Si 2p core regions of SiMg2.5 and SiMg5.0. (**c**) TGA curves of SiMg2.5, SiMg5.0, and SiMg7.5 measured in air. The numbers in the graph indicate the ratio of weight gain (wt%) and Si mole fraction (*n*_Si_/(*n*_Si_ + ${{n}_{{\mathrm{SiO}}_{2}}}$)).

Figure 4a shows the X-ray diffraction (XRD) patterns of SiMg2.5 before HCl etching and SiMg2.5, SiMg5.0, and SiMg7.5 after HCl etching. Diffraction peaks that were attributed to Si and MgO were observed before HCl etching. The diffraction peaks of MgO were absent in the XRD patterns of SiMg2.5, SiMg5.0, and SiMg7.5 after HCl etching; additionally, the remaining main peaks were indexed to cubic Si. The Si phase and the halo patterns of SiO_x_ were detected in SiMg2.5. This indicated that the lack of Mg vapor resulted in insufficient reduction. Small amounts of Mg silicates, such as MgSiO_3_ and Mg_2_SiO_4_, were also detected in SiMg2.5, SiMg5.0, and SiMg7.5. The formation of Mg silicates was due to insufficient Mg vapor pressure, especially in the interfacial region of SiO_2_ and MgO within the fibers^32,39^, and the local high-temperature environment caused by the rapid exothermal reaction. Magnesiothermic reduction under vacuum promotes the generation of Mg vapor, which is prone to escape from the reaction system. Therefore, controlling the Mg vapor pressure is critically important for the fabrication of pure Si. Although Mg silicates may negatively affect the electrochemical performance of a material, their high elastic modulus can inhibit internal cracking owing to the electrochemical agglomeration of nanometer-sized Si^40^.

The oxygen contents on the surfaces of SiMg2.5 and SiMg5.0 can be compared using the Si 2p core-level X-ray photoelectron spectroscopy (XPS) profiles shown in Fig. 4b. The peaks at approximately 99, 101, and 104 eV of SiMg2.5 were attributed to Si, Si-Si_n_O_4−n_ (n = 1–3), and SiO_2_, respectively. Regarding SiMg5.0, the increase in peak intensity at 99 eV was attributed to Si, the absence of the peak at 104 eV was attributed to SiO_2_ depletion, and the presence of peaks at 103–100 eV were attributed to Si-Si_n_O_4−n_ (n = 1–3). This indicated that the oxygen content was reduced. Thermogravimetry/differential thermal analysis (TG/DTA) measurements were performed to investigate the Si mole fraction (*n*_Si_/(*n*_Si_ + ${{n}_{{\mathrm{SiO}}_{2}}}$)) in the nanoporous Si fibers (Fig. 4c). The weight gain of the fibers corresponded to the weight of oxygen required to oxidize Si to SiO_2_. The Si mole fractions of SiMg2.5, SiMg5.0, and SiMg7.5 were 0.44, 0.83, and 0.72, respectively. The obtained oxygen contents corroborated the XRD and XPS results. In subsequent experiments, we focused on the applications of SiMg5.0, which had the highest Si mole fraction and porosity among the samples, to all-solid-state batteries.

### Performance of nanoporous Si fibers in all-solid-state batteries

**Figure 5:**
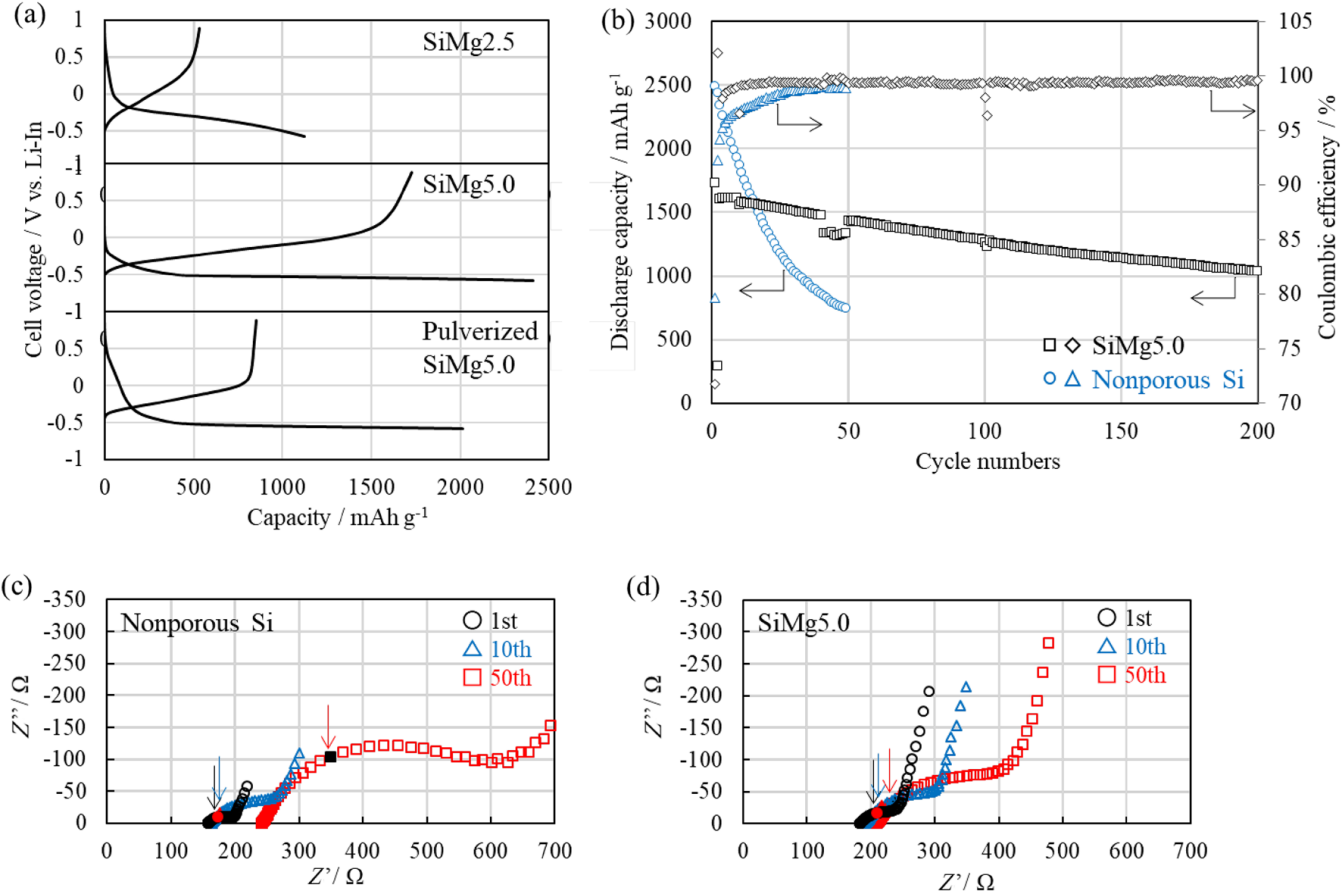
Effect of morphology and Si content on the electrochemical performance of the nanoporous fibers. (**a**) Initial charge/discharge curves of SiMg2.5, SiMg5.0, and pulverized SiMg5.0. (**b**) Cycling stability of anodes based on SiMg5.0 and nonporous Si. (**c**, **d**) Nyquist plots of (**c**) nonporous Si and (**d**) SiMg5.0 after charging for 1st, 10th, and 50th cycles. The arrows indicate the position at a frequency of 1 Hz.

The performance of the nanoporous Si fibers was verified via electrochemical characterization. The initial charge–discharge curves of SiMg2.5 and SiMg5.0 are shown in Fig. 5a (top, middle). SiMg2.5 exhibited initial charge/discharge capacities of 1124/530 mAh g^−1^, with an ICE = 47% (Fig. 5a top, Table S3). The initial charge capacity was lower than the theoretical capacity of Si (Li_15_Si_4_, 3572 mAh g^−1^) because of the high oxygen content with a Si fraction of 0.44 (theoretical capacity of SiO is ~ 1710 mAh g^−1^^41^) (Fig. 4b,c). In addition, Si embedded in the SiO_2_ matrix may be affected by the strong compressive stress during lithiation, which limits the charging capacity^42^. A high oxygen content also results in a low ICE, because Li^+^ is irreversibly trapped as lithium silicates, Li_4_SiO_4_, and Li_2_O via the conversion reaction of Li^+^ with SiO_x_ at the initial charge^43^. In addition, by-products, such as MgSiO_2_ and Mg_2_SiO_4_ (Fig. 4a), irreversibly react with Li^+^ to produce Li_2_SiO_2_ and Li_4_SiO_4_. In comparison, SiMg5.0 achieved initial charge/discharge capacities of 2411/1729 mAh g^−1^, corresponding to a relatively high ICE of 71% (Fig. 5a middle, Table S3). The improved capacities and ICE could be attributed to its low oxygen content with a Si fraction of 0.83 (Fig. 4b,c).

To demonstrate the importance of the fiber structure to the performance of the anode, we crushed SiMg5.0 using an agate mortar and pestle under a dry Ar atmosphere to produce samples with a small aspect ratio (diameter of approximately 500 nm, as measured by SEM). This sample was referred to as pulverized SiMg5.0. The fiber morphology of SiMg5.0 was roughly preserved in the composite powder prepared by mixing SiMg5.0, SE and AB for 3 min (Fig. S5a) and in the corresponding composite anode prepared by pressing at 333 MPa (Fig. S5b). The surface oxide film, SiO_x_, and by-products, MgSiO_2_ and Mg_2_SiO_4_, may provide some mechanical strength to the fibers. In contrast, the SiMg5.0 fiber morphology was not observed in the composite powder prepared by mixing pulverized SiMg5.0, SE and AB in an agate mortar and pestle for 5 min (Fig. S5c). Pulverized SiMg5.0 exhibited initial charge/discharge capacities of 2017/853 mAh g^−1^ and ICE of 42%, which were lower than those of the original SiMg5.0 (Fig. 5a bottom, Table S3). The reduction in initial charge capacity indicates that the number of isolated Si particles increased without connected electronic and ionic pathways constructed by SE and AB, resulting in lower Si utilization. The reduction in ICE could be attributed to the fact that the connections of the electronic and ionic conduction pathways are easily severed by even a marginal volume reduction in the pulverized SiMg5.0 during initial delithiation. The pulverized SiMg5.0 exhibited a stable cycle performance after two cycles, as discussed below (Fig. S6). Therefore, the main role of the fibrous structure was to prevent the isolation of Si during initial charge and discharge. SE and AB are major factors governing the fabrication of the ionic and electronic conduction pathways in the composite anode. Compared with pulverized SiMg5.0, SiMg5.0 can be at least partly in contact with SE and AB. The lithiation of SiMg5.0 begins at the contact interface, increasing the Li^+^ diffusion coefficient (~ 10^−12^ cm^2^ s^−1^, as estimated by Eq. S1) and electronic conductivities^21–23^. Therefore, the partially lithiated Si assists Li^+^ diffusion and electron transport through the fibrous structure, even when contact between the Si fiber and SE/AB is partially lost, thus improving Si utilization.

The cycling performances of the porous and nonporous structures were compared (Fig. 5b). The Coulombic efficiency (CE) of SiMg5.0 increased to over 99.0% after 11 cycles. After 40 cycles, SiMg5.0 maintained a discharge capacity of 1474 mAh g^−1^ and capacity retention of 85%. The active material loading (0.9 mg cm^−2^) provided an areal capacity of approximately 1.3 mAh cm^−2^ after 40 cycles. SiMg5.0 exhibited stable cycle performance, achieving a capacity of 1038 mAh g^−1^ after 200 cycles with a capacity retention of 60%. The expansion volume calculated from the reversible capacity can be compensated by the pore volume of SiMg5.0, which indicates negligible outward expansion. In addition, the small amounts of lithium silicate and Li_2_O formed during the initial charge enhanced the mechanical properties of the fibers and inhibited SE decomposition. Pulverized SiMg5.0 also exhibited stable cycle performance with a capacity of 800 mAh g^−1^ after 100 cycles and a capacity retention of 83% compared with that of the maximum discharge capacity (Fig. S6). Therefore, the cycling of pulverized SiMg5.0 particles that could retain ionic and electronic conduction pathway connections during initial charge/discharge performed stably thereafter. This suggests that the porous structure prevented significant outward volume change during repeated cycling.

Compared with nanoporous Si, nonporous Si powders with diameters of 1–5 μm exhibited higher initial charge/discharge capacities of 3121/2487 mAh g^−1^ with an ICE of 80% (Table S3). This occurred because of the low specific surface area of nonporous Si powders, which resulted in a low oxygen content. However, a drastic reduction in capacity was observed in subsequent cycles. CE did not exceed 99.0% until the 40th cycle; additionally, it exhibited a capacity retention of only 35% after 40 cycles. Although SiMg5.0 with half density of nonporous Si resulted in a low volumetric capacity, the volumetric capacity of SiMg5.0 exceeded that of nonporous Si at approximately the 50th cycle. To determine the reason behind the difference in the cycling stability of the fibers, we compared the change in the internal resistance of the cells during cycling.

Figures 5c,d show the results of the electrochemical impedance spectroscopy (EIS) measurements of nonporous Si and SiMg5.0 after the 1st, 10th, and 50th charges. An equivalent circuit shown in Fig. S7 was used to fit the EIS spectra. The intercept values on the x-axis at frequencies above 10 kHz can be attributed to the resistance of the bulk and grain boundary for the SE layer, i.e., the R_SE_. An arc in the middle-frequency region with a peak frequency of 1 Hz can be attributed to the charge transfer resistance, R_ct_, of the interface in the Si/SE^24^. The straight line in the low-frequency region (lower than 0.01 Hz) is attributed to Li^+^ diffusion in the active materials, i.e., Warburg impedance. The R_SE_ and R_ct_ values of nonporous Si and SiMg5.0 increased with cycling. The impedance growth rate relative to the corresponding impedance after the initial charge was used to compare the nonporous Si and SiMg5.0. This was used because these structures cannot be directly compared using absolute values. In particular, R_ct_ reflects the differences in the types and quantities of surface products, such as Li_2_O, with low ionic conductivity (~ 10^−9^ S cm^−1^) formed at the initial charge and the contact area at the Si/SE interface. The R_SE_ growth rate of nonporous Si after the 50th cycle increased 1.5-fold, which was larger than that of SiMg5.0 (1.1-fold). The increase in R_SE_ indicates that the contact between SE particles was loose^25,44^ and cracks were formed in the SE layer. The R_ct_ growth rate of SiMg5.0 after 50 cycles increased approximately three-fold but was lower than that observed for the nonporous Si (approximately eight-fold). The difference in the R_ct_ growth rate between the nanoporous Si fibers and nonporous Si indicates that there was a change in the contact area at the Si-SE interface. In particular, SiMg5.0 can maintain close contact with the surrounding SE, whereas the nonporous Si cannot.

**Figure 6:**
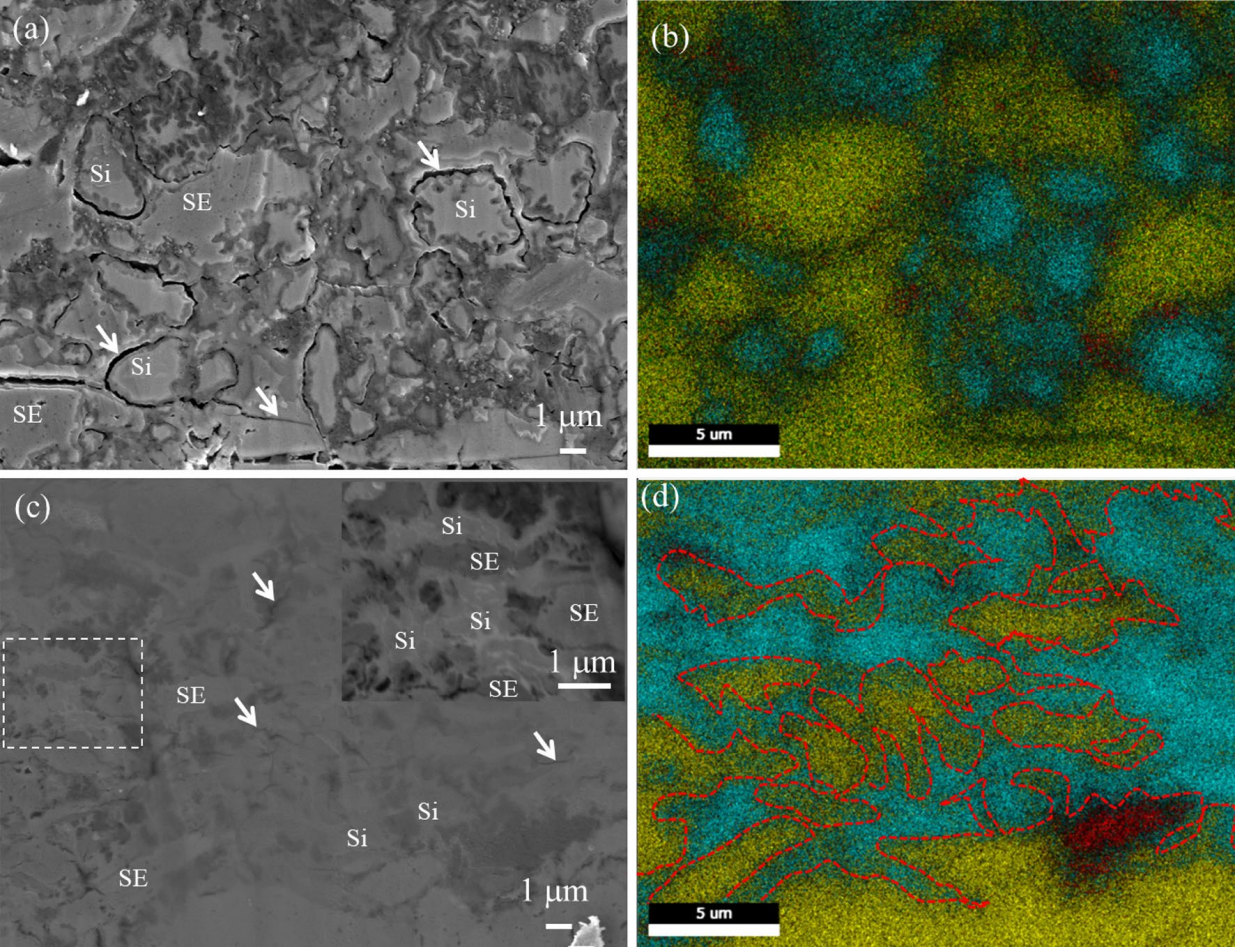
Comparison of the morphologies of composite electrodes based on the nanoporous Si fibers and nonporous Si after 50 cycles. (**a**, **c**) Cross-sectional SEM images and (**b**, **d**) corresponding EDS elemental maps of composite electrodes based on (**a**, **b**) nonporous Si and (**c**, **d**) SiMg5.0. Si = light blue, S = yellow, P = green, C = red. The arrows in (**a**, **c**) indicate cracks. White dotted box in (**c**) represents the enlarged region shown in the inset. The red dotted lines in (**d**) represent the network structure of the nanoporous Si fibers.

To visualize the morphology change of the composite anodes during cycling, we obtained the cross-sectional SEM–EDX images of SiMg5.0 and nonporous Si. In nonporous Si, horizontal cracks surrounding the composite anodes/SE layer interface and inside the SE layer after the 50th cycle were observed (Fig. S8b). This indicates that the large expansions of the nonporous Si composite anode induced compression of the SE layer, leading to elastic deformation and then plastic deformation of the SE layer. Subsequently, it was challenging to conform the plastically deformed SE layer to the anode contractions. The repeated expansions and contractions of the anode induced compression and tension in the SE layer, thus forming horizontal cracks. Large cracks and gaps were also observed between the nonporous Si and surrounding SE in the composite anode after the 50th cycle (Fig. 6a,b). During lithiation, the large outward expansion of the nonporous Si causes elastic deformation of the surrounding SE, followed by plastic deformation. Subsequently, the SE is unable to conform to the shrinkage of Si during delithiation, resulting in the formation of cracks between Si and the SE. Poor contact at the SE layer and nonporous Si/SE interface lead to increased R_SE_ and R_ct_ growth rates, respectively, and significant capacity decay.

Composite anodes containing SiMg5.0 adhered to the SE layer (Fig. S8d) and exhibited few microscopic cracks (Fig. 6c) after the 50th cycle. We focused on the morphology change of fibers in the composite anode. The boundaries of Si fibers and the voids in the composite anode were clearly observed before initial cycling (Fig. S9a). After the first lithiation, the boundaries of Si fibers and the voids were absent; additionally, no cracks were observed (Fig. S9b). This suggests that SiMg5.0 expanded outward and adhered closely to the surrounding Si, SE, and AB. If the total pore volume of SiMg5.0 was used as buffer space by pore shrinkage, the outward expansion of SiMg5.0 at initial charge capacity, 2411 mAh g^−1^ (Li_2.5_Si), corresponded to V/V_0_ =  ~ 1.8^45^. If the pores expand back to the original total pore volume, subsequent delithiation will result in a minimal variation in the outward volume as estimated from the initial discharge capacity of 1729 mAh g^−1^. During 50 cycles, deformation of the fibers in the composite anode to that of an ameba-like shape occurred (Fig. 6c inset). This entangled with the surrounding SE and connected to other fibers, forming an interconnected network throughout the dense electrode (Fig. 6c,d). Although the pristine Si fibers did not deform, even under high pressure (333 MPa), during cell fabrication (Fig. S5b), the hardness of the Li-Si alloys decreased significantly during lithiation; Young’s modulus of pristine Si (90 GPa) decreased to less than 40 GPa for Li_3.75_Si^46^. Therefore, Li_x_Si fibers plastically deformed under high constraint pressures to an ameba-like shape and formed interconnected network structures during repeated charge and discharge. Even if the fibers experienced small outward volume changes, the sulfide-based SE can elastically deform to some extent^47^, thereby maintaining close interfacial contact. These observations are congruent with the stable cycling performance and low growth rate of R_SE_ and R_ct_. Therefore, the porous and network structures of the fibers assisted in the formation and maintenance of ionic and electronic conduction pathways, thereby enabling high capacities and reversibility.

## Conclusions

We developed a composite anode with an interconnected network of nanoporous Si fibers. The fibers were synthesized via a simple, scalable, and cost-effective method. A relatively low oxygen content of 0.83 Si mole fraction was achieved without hydrofluoric acid treatment by controlling the synthesis conditions. Low oxygen contents guaranteed low irreversible Li loss owing to silicon oxide lithiation. Moreover, small amounts of lithium silicate and Li_2_O enhanced the mechanical properties of the fibers and protected the surrounding SE from reductive decomposition. The total pore volume (0.519 cm^3^ g^−1^) corresponded to a volume change of 1635 mAh g^−1^ in specific capacity. Therefore, it is estimated that the sample undergoes no outward volume change during repeated cycling.

The effects of the porous and fibrous structures of nanoporous Si fibers were exhibited by comparing the charge/discharge performance via EIS and SEM analyses of cells containing pulverized nanoporous Si and nonporous Si. The porous structure of the fibers effectively accommodated volume expansion into the pores, resulting in stable cycle performance owing to the maintenance of close contact between Si and the SE/AB. The fibrous structure can easily form a network, and the lithiated Si fiber network provides auxiliary pathways through which Li ions and electrons can be conducted. This network also compensated for the partial interfacial disconnection of ionic and electronic conduction pathways constructed by SE and AB, particularly during initial cycling, leading to improvements in Si utilization and ICE. The composite anode exhibited a stable reversible capacity of 1474 mAh g^−1^ with a capacity retention of 85% after 40 cycles and achieved a capacity of 1038 mAh g^−1^ with a capacity retention of 60% after 200 cycles. After 40 cycles, active material loading (0.9 mg cm^−2^) provided an areal capacity of approximately 1.3 mAh cm^−2^, which was similar to the values of commercial Li-ion batteries. To maintain interfacial contact, high constraint pressures must be generally applied to all-solid-state batteries, especially those with Si anodes which undergo large volume changes during charging and discharging^25^. The proposed composite anode has the potential to reduce constraint pressure; therefore, it may apply to large-area batteries. For future studies on mechanical properties, we intend to gain insights into the effect of constraint pressures applied to full-cells, as well as the changes in thickness and pressure during charge and discharge. Further research to improve ionic and electronic conductivities and mechanical properties of the nanoporous Si fibers by the structural and interfacial design using carbon^48–51^ and SE coating^47,52^ can lead to enhanced cycling and rate performances. Overall, the proposed approach provides significant advantages for designing high-capacity anodes suitable for all-solid-state batteries.

## Methods

### Nanoporous Si fiber preparation

Nanoporous Si fibers were fabricated by electrospinning, followed by magnesiothermic reduction (Fig. S10), according to previous reports^53^. TEOS (Nacalai, 1.5 g) and deionized water (4 g) were stirred at room temperature for 10 min, added with PVP (Alfa Aesar; Mw: 1,300,000, 1 g) and ethanol (3.5 g), and mixed for 15 min using a mixer (KURABO KK-250S). Acetic acid (1 g) was added to the solution, which was subsequently mixed for 3 min and stabilized for 30 min to obtain the spinning precursor. The precursor solution was electrospun at an ambient temperature of 28 °C and less than 45% humidity using an electrospinning machine (Katotech) equipped with a syringe and static corrector. A voltage of 15 kV was applied between the tip of the syringe and the collector at a horizontal distance of 15 cm.

The resulting TEOS/PVP nonwoven fiber sheets were placed in a ceramic boat in a furnace, calcined in air at a ramp rate of 10 °C/min from 30 °C to 700 °C, and maintained at 700 °C for 2 h to remove organic components. Calcined SiO_2_ fiber sheets and Mg (Wako, 40 mesh) with molar ratios of 1:x (x = 2.5, 5.0, 7.5) were alternately layered in an alumina boat. The boat was placed in a steel cylinder and assembled in an Ar-filled glovebox. The steel container was placed in a tubular furnace, heated at a ramp rate of 10 °C/min to 685 °C, and held at 685 °C for 45 min under vacuum. The obtained Si/MgO fibers were soaked in 1 M HCl for 4 h for MgO and Mg_2_Si removal and then filtered. The dark brown precipitate was washed with water and ethanol, collected via filtration, and dried for 12 h at room temperature under a vacuum. Washing was performed in an Ar-filled glovebag. The HCl, water, and ethanol were degassed with Ar before washing.

### Material characterization

The fiber microstructures were observed using FE-SEM (JSM-7800F, JEOL). The chemical structure was analyzed using XRD (SmartLab, Rigaku) with Cu-Kα radiation and XPS (Kratos AXIS-ULTRA, SHIMADZU). Specific surface areas and pore distributions were determined using a Brunauer–Emmett–Teller analyzer (ASAP2020. Shimadzu). TG/DTA measurements (STA2500, Regulus, Netzsch) were performed at a heating rate of 10 °C/min to 1000 °C (held at 1000 °C) in air.

### Half-cell fabrication and characterization

All synthesis, fabrication, and testing steps were performed in a dry Ar-filled glovebox.

Two types of 75Li_2_S·25P_2_S_5_ glass SEs were prepared according to previous reports. Rough SE was prepared from Li_2_S (Furuuchi Chemical) and P_2_S_5_ (Sigma-Aldrich) by mechanical milling (ϕ ~ 10 μm, ionic conductivity: 5 × 10^−4^ S cm^−1^) and used as the SE layer^54^. Fine SE (ϕ1–3 μm, ionic conductivity: 2 × 10^−4^ S cm^−1^) was prepared by pulverizing rough SE and used as the composite anode material^55^.

Nanoporous Si fibers (15 mg), fine SE (25 mg), and AB (Denka, 3.75 mg) were mixed at a weight ratio of 40:60:10 using an agate mortar and pestle for 3 min with weak force to maintain the fiber shape, resulting in a homogeneous brown powder as anode composite (Fig. S5a). To fabricate the half-cells (Fig. S11), we placed the rough SE powder (80 mg) in a polyetheretherketone (PEEK) holder (diameter, 10 mm) and pressed it between stainless-steel rods at 37 MPa to form a SE layer. The anode composite (2 mg) was spread onto the side of the SE layer and pressed at 333 MPa to form a two-layered pellet. Li (ϕ5 × 0.2 mm)–In (ϕ8 × 0.1 mm) foil, as the counter electrode, was placed on the opposite side of the SE layer and pressed under a pressure of 111 MPa. The cells were sandwiched between two stainless-steel rods and held with bolts and nuts. For comparison, anode composites with metallurgical-grade Si (1–5 μm, Alfa Aesar) and pulverized SiMg5.0, pre-milled using an agate mortar and pestle with strong force (Fig. S5c), were prepared by mixing Si, fine SE, and AB using an agate mortar and pestle for 5 min.

Galvanostatic cycling was conducted at 0.100 mA cm^−2^ (0.026 C) at 30 °C using a charge/discharge measurement device (BTS-2004, Nagano). The current density was selected as the condition that allows active materials appropriate evaluation, due to avoid large polarization for solid-state batteries using LPS glass with a relatively low ionic conductivity of ~ 10^−4^ S cm^−1^. The voltage cutoffs were 0.88 and − 0.58 V vs. Li–In, corresponding to voltages of 1.5 and 0.04 V vs. Li/Li^+^, respectively. Regarding cycle performance of SiMg5.0 (Fig. 5b), a sudden drop in capacity in the 2nd cycle was due to the cable disconnecting, not a cell fault. In addition, the drop in capacity from the 41st to 50th cycles was owing to power failure, which resulted in a drop in temperature in the thermostatic chamber. Specific capacities were calculated from the Si mass. The C rate was calculated by assuming a 4200 mAh g^−1^ theoretical capacity for Si. Si lithiation and delithiation in the half-cells were defined as the charging and discharging steps, respectively. EIS measurements were performed at an amplitude of 50 mV and a frequency range of 2 mHz–1 MHz at 30 °C using an AC impedance analyzer (FRA1455, Solartron) after charging to − 0.58 V.

To characterize the microstructures of the electrodes after cycling, we prepared cross-sections of the specimens by Ar-ion milling using a cross-sectional polisher (CP, 1B 19520CCP, JEOL) and observed them under an FE-SEM equipped with an energy dispersive X-ray spectrometer (TEAM EDS, EDAX). The samples were transferred from an Ar-filled glovebox to the CP and FE-SEM instrument using a transfer vessel without exposure to air.

### Supplementary Information

Supplementary Information.

## Data Availability

The datasets used during the current study available from the corresponding author on reasonable request.
