# Supplementary material for: Nanoporous silicon fiber networks in a composite anode for all-solid-state batteries with superior cycling performance
Source: Sci Rep. 2023 Oct 10;13:17051. doi: 10.1038/s41598-023-44070-1 (PMC10564847; doi:10.1038/s41598-023-44070-1)
Supplement: Supplementary file 1 — Supplementary Information. [file 41598_2023_44070_MOESM1_ESM.docx]

Supplementary

**Nanoporous silicon fiber networks in a composite anode for all-solid-state batteries with superior cycling performance**

**Mari Yamamoto*^a,b^*, Mika Takatsu*^b^*, Ryota Okuno*^b^*, Atsutaka Kato*^a^*, Masanari Takahashi*^a,b^***

*^a^*Osaka Research Institute of Industrial Science and Technology, Morinomiya Center, 1-6-50, Morinomiya, Joto-ku, Osaka-city, Osaka 536-8553, Japan

*^b^* Graduate School of Materials Science, Nara Institute of Science and Technology, 8916-5 Takayama-cho, Ikoma, Nara 630-0192, Japan

* Tel.: +81-6-6963-8085. Fax: +81-6-6963-8099. E-mail: yamamoto.mari@orist.jp


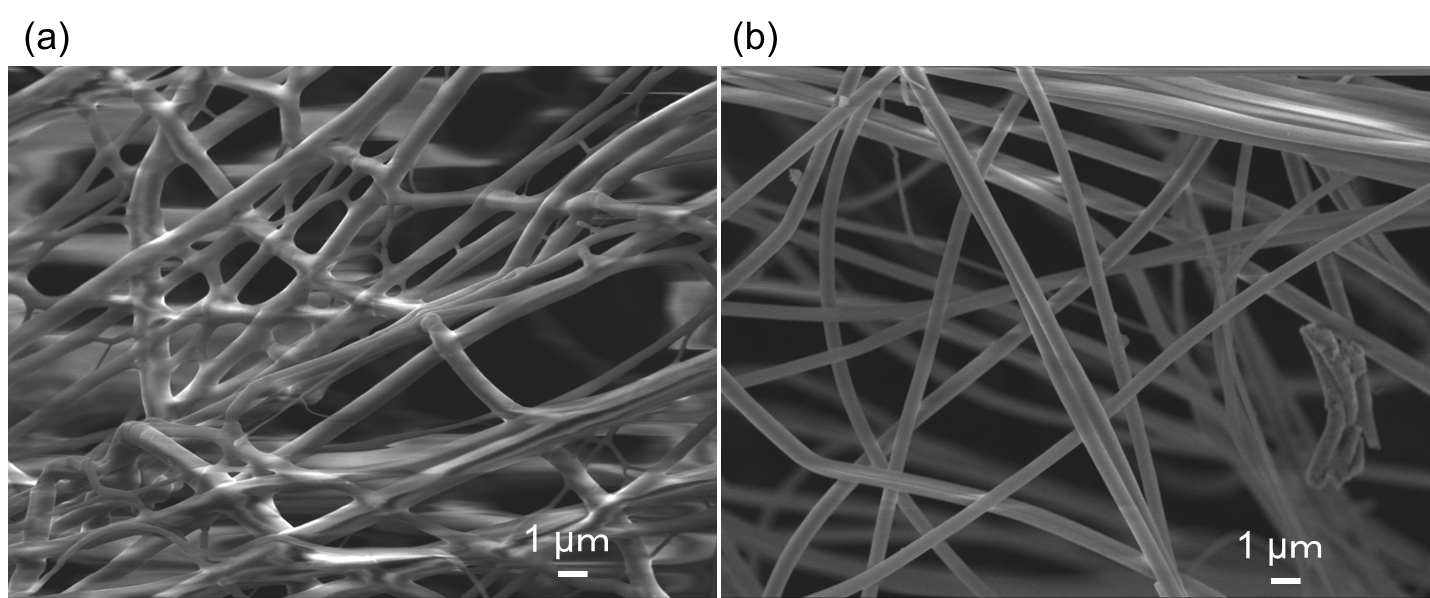


**Figure S1.** SEM images of as-spun fibers prepared at relative humidity; (**a**) approximately 50% and (**b**) below 45%.


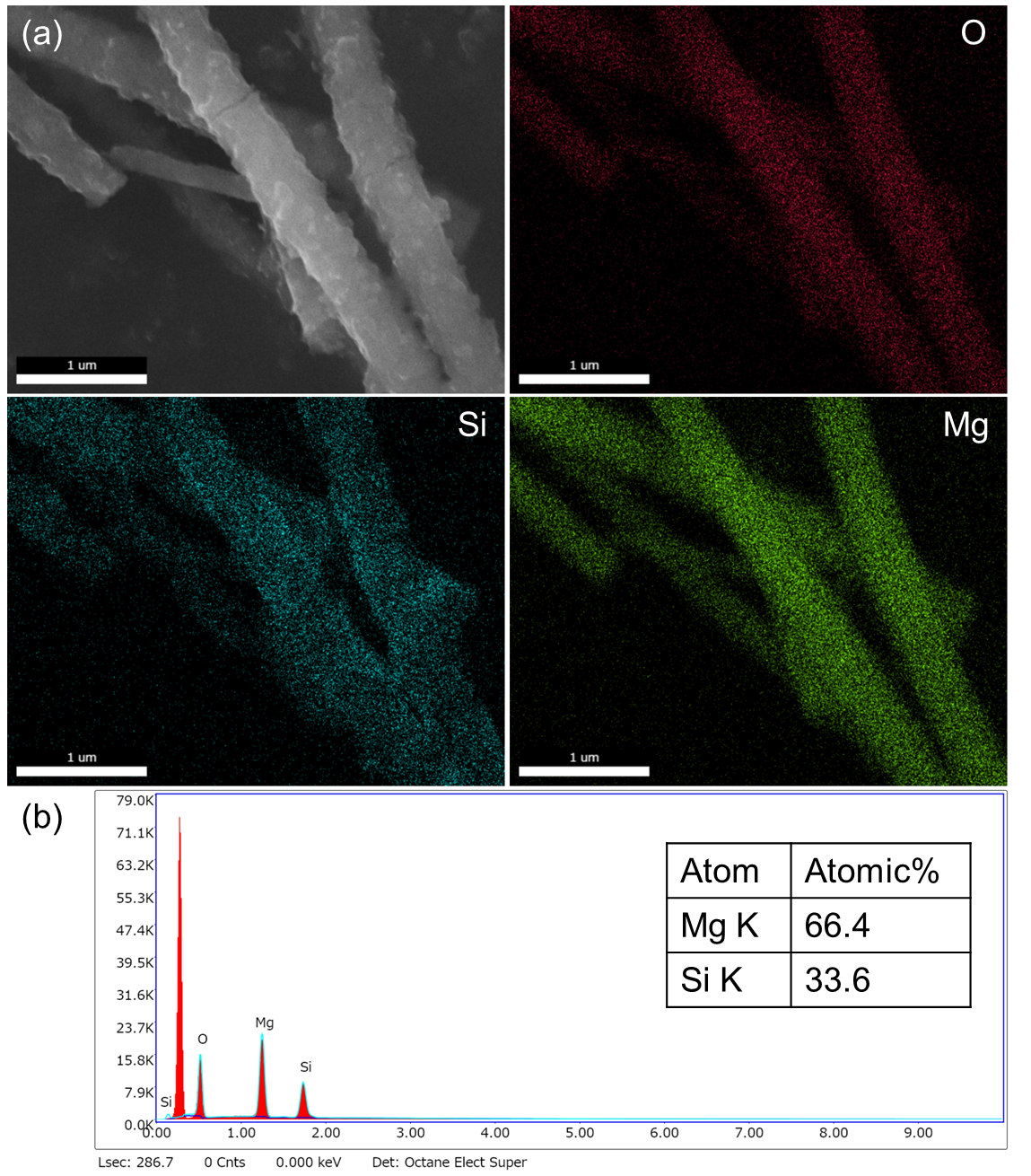


**Figure S2.** (**a**) SEM image of SiMg5.0 before HCl etching and the corresponding EDX elemental maps for Si (light blue), Mg (green) and O (red). (**b**) The corresponding EDX analysis; the inset table displays the atomic% of the elements.


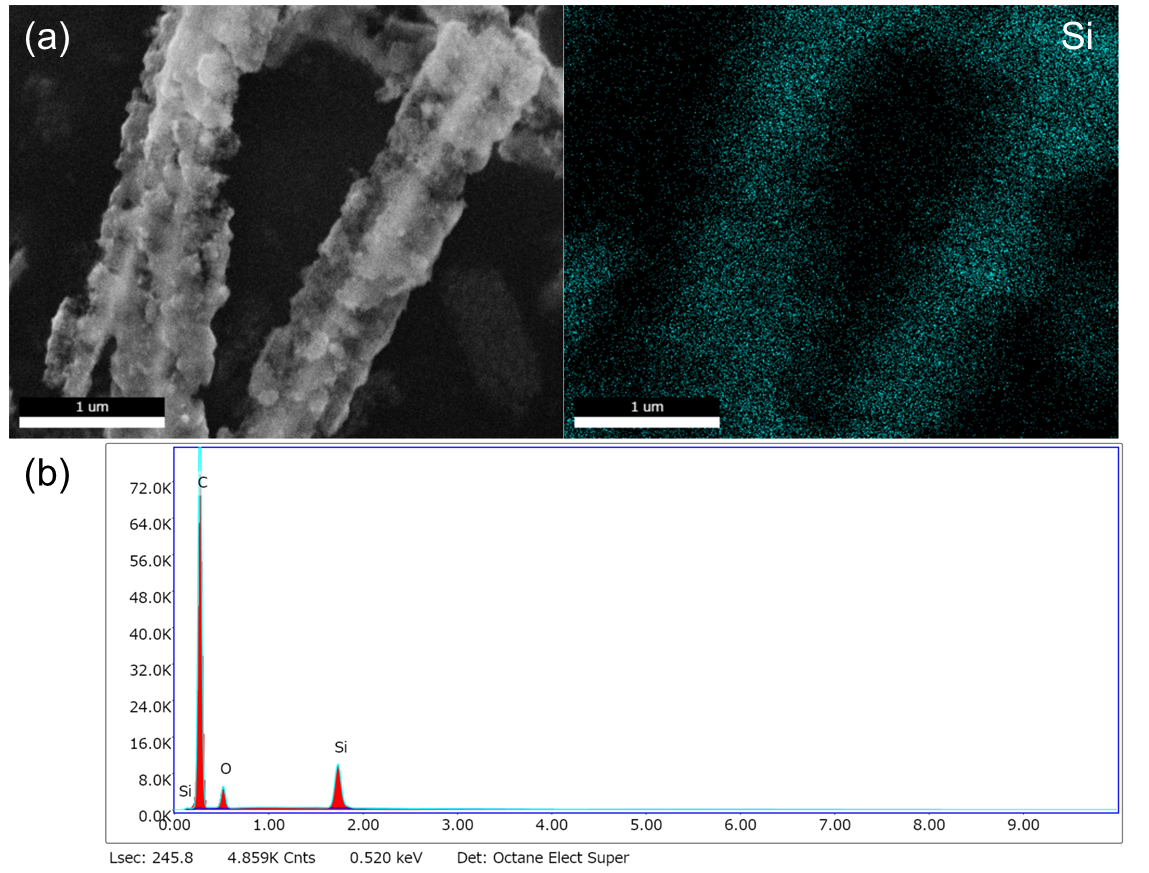


**Figure S3.** (**a**) SEM image of SiMg5.0 and the corresponding EDX elemental map for Si (light blue). (**b**) The corresponding EDX analysis.


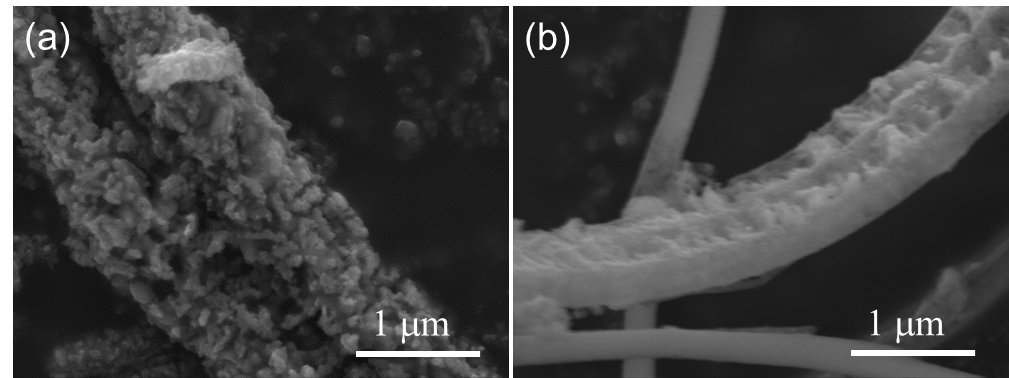


**Figure S4.** SEM images of (**a**) SiMg7.5 and (**b**) SiMg2.5.


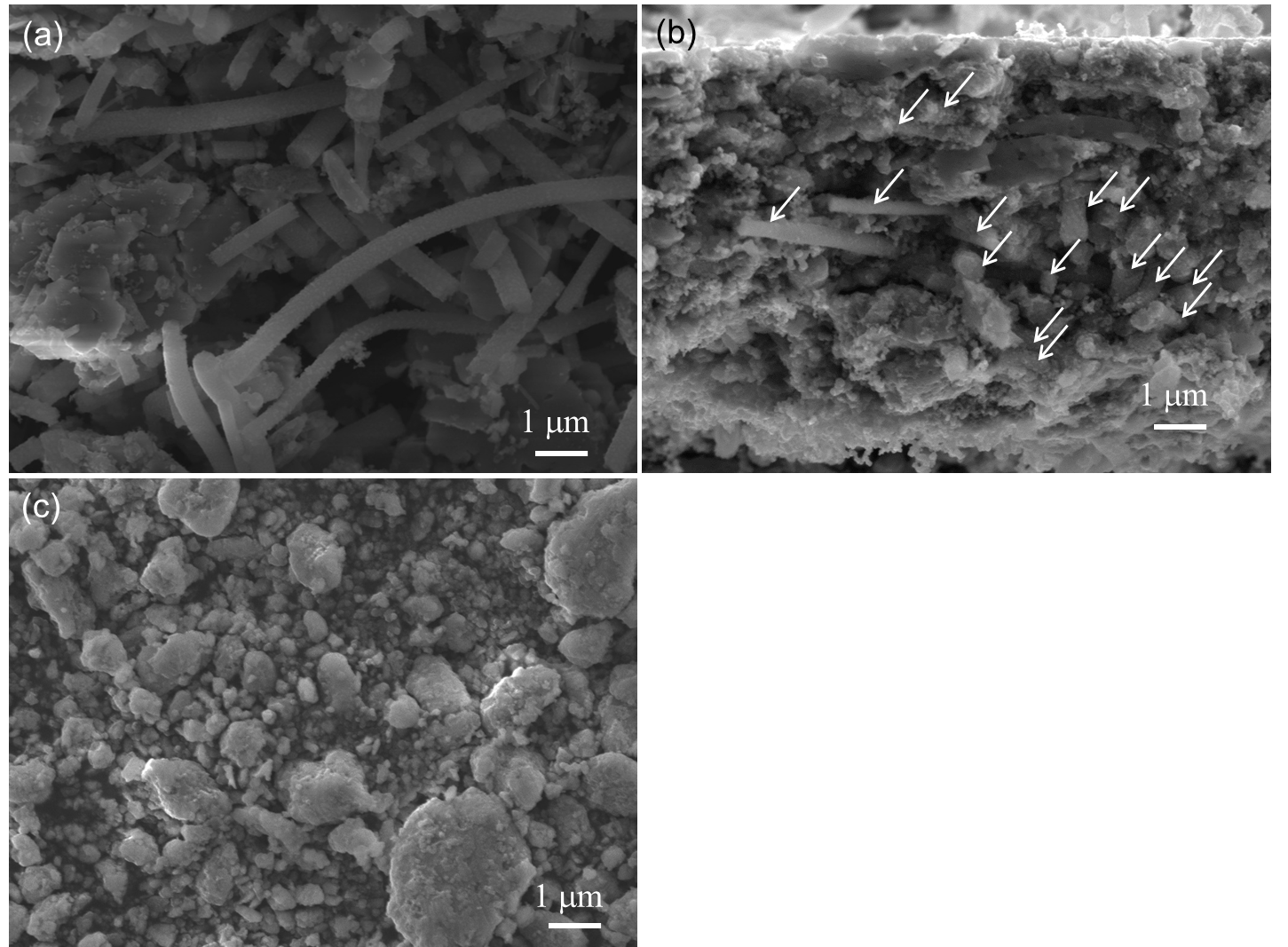


**Figure S5.** SEM images of (**a**) the composite powder prepared by mixing SiMg5.0, SE and AB using an agate mortar and pestle for 3 min with weak force. (**b**) The cross-sectional fracture surface of the corresponding composite anode prepared by pressing at 333 MPa. Arrows indicate SiMg5.0 fibers. (**c**) The composite powder prepared by mixing pulverized SiMg5.0, SE and AB using an agate mortar and pestle for 5 min. The pulverized SiMg5.0 was prepared in advance by strong force grinding using an agate mortar and pestle.


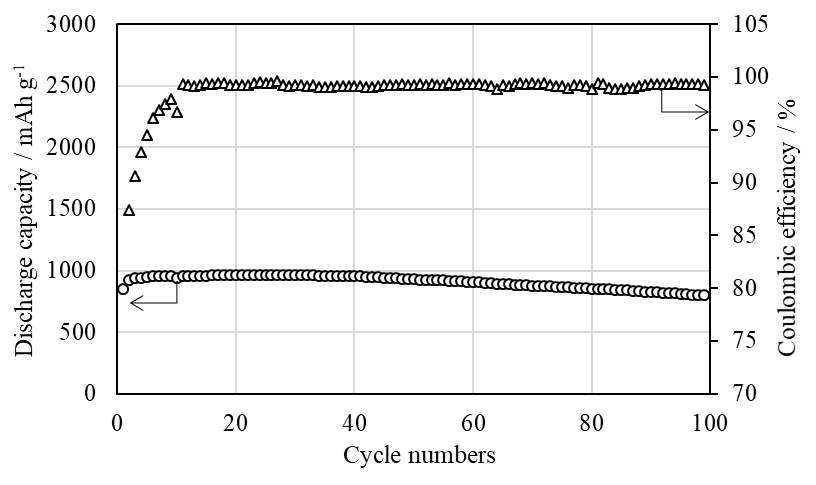


**Figure S6.** Cycling stability of anodes based on pulverized SiMg5.0.


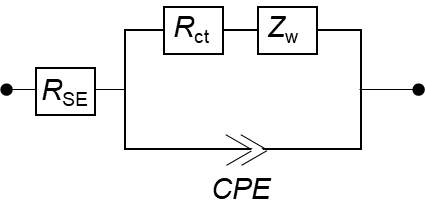


**Figure S7.** The equivalent circuit used to fit the EIS spectra in Fig. 5c, d. The resistance of the counter electrode, Li-In/SE interface was excluded because it was found to be negligible from previous EIS results [23, 24].


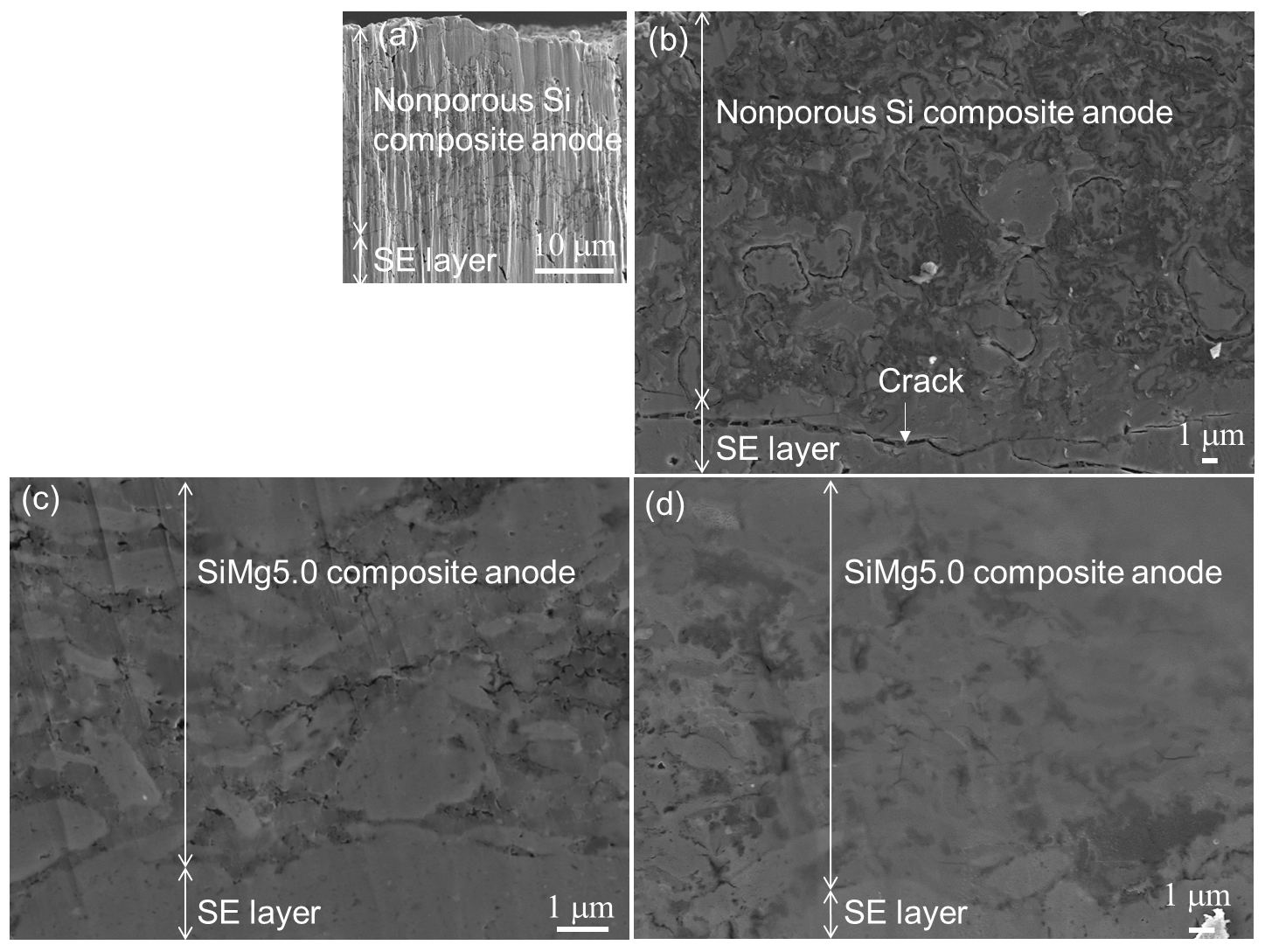


**Figure S8.** Cross-sectional SEM images for the interfacial structure of (**a**, **b**) nonporous Si composite anode/SE layer and (**c**, **d**) SiMg5.0 composite anodes/SE layer (**a**, **c**) before cycling and (**b**, **d**) after the 50^th^ cycle.


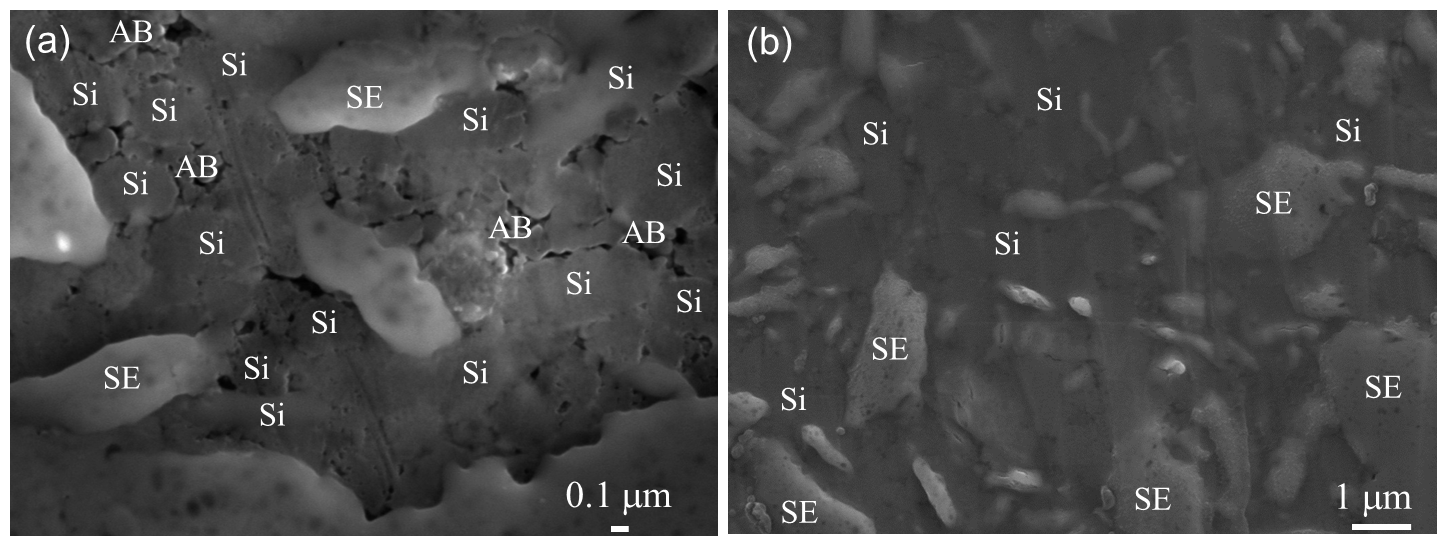


**Figure S9.** Cross-sectional SEM images of SiMg5.0 composite anodes (**a**) before cycling and (**b**) after the first lithiation.


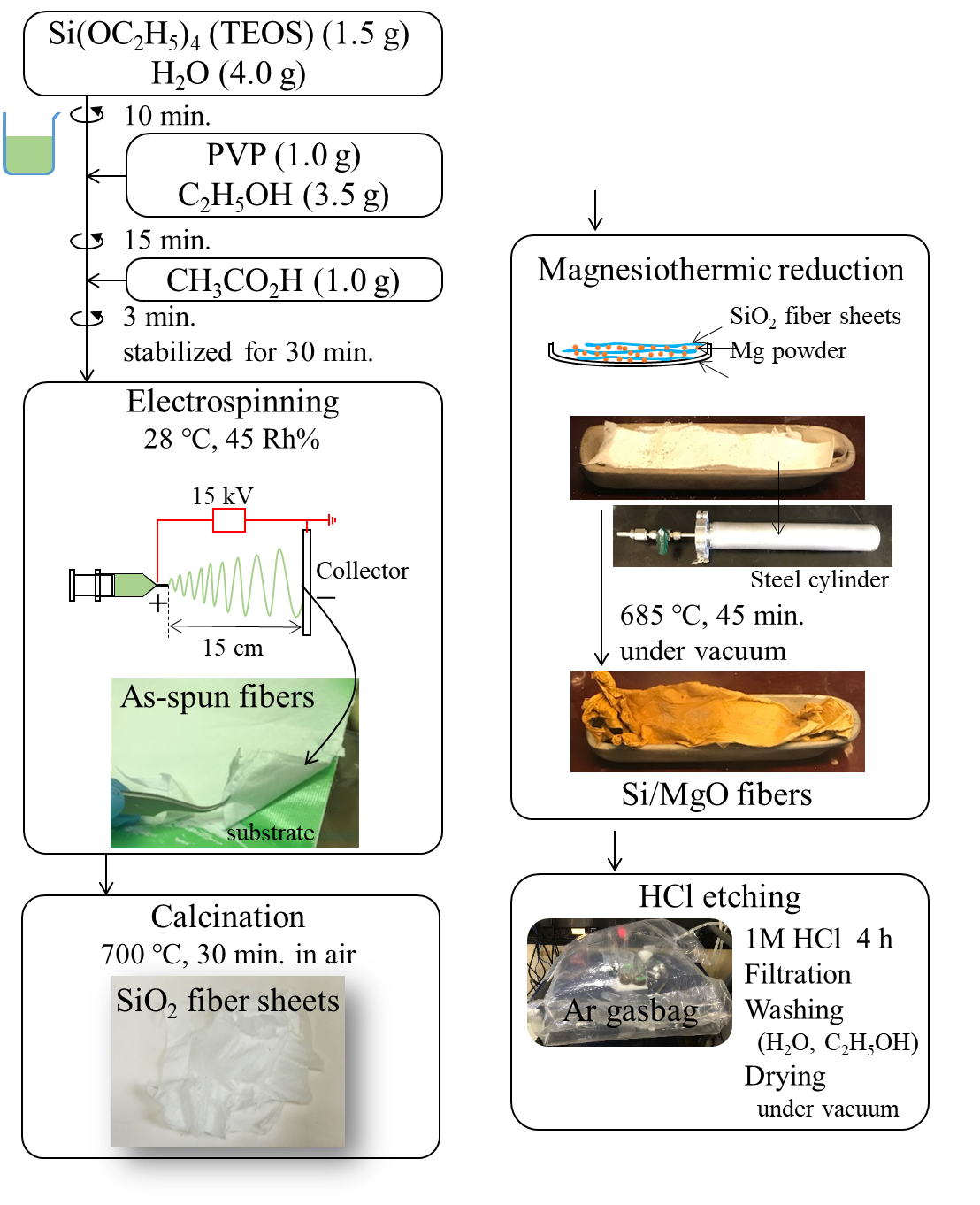


**Figure S10.** Schematic of the nanoporous Si fiber synthesis process and photographs of the intermediate products.


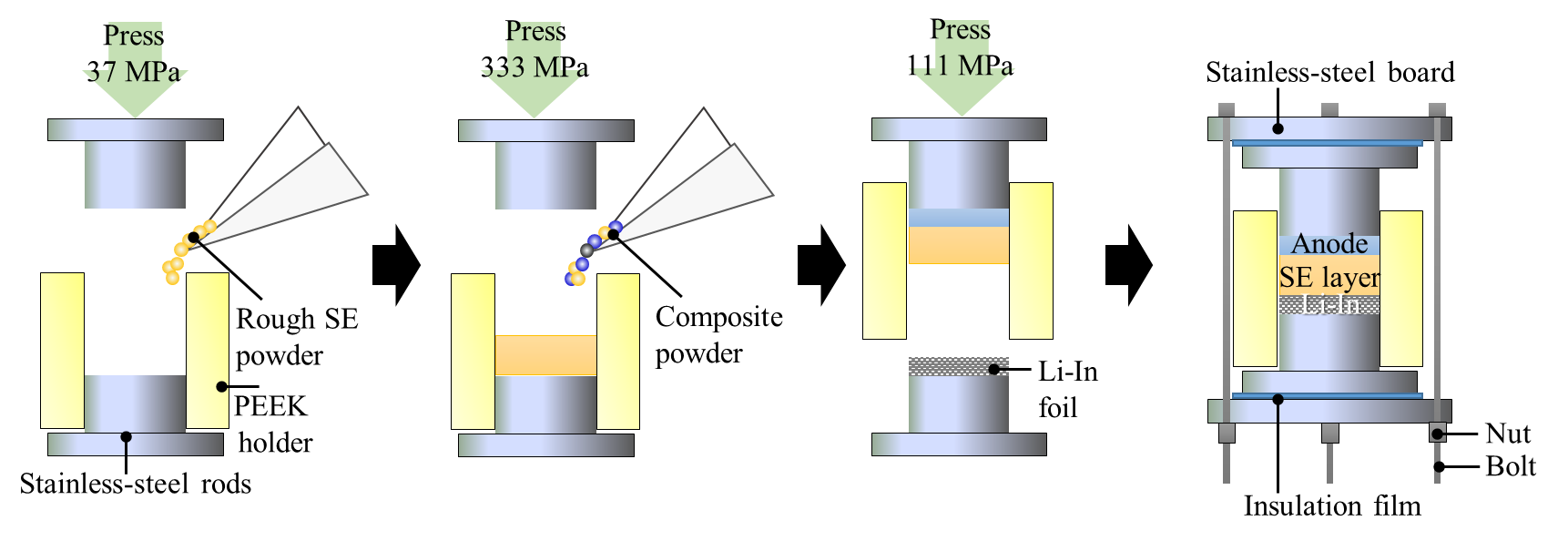


**Figure S11.** Schematic of cell fabrication and showing the tightening of the cell by the cell holder using bolts and nuts at three points.

**Table S1.** Energy densities of all-solid-state batteries comprising Si anodes and graphite anodes.

|  | Graphite/NCM811 full cell | Si/NCM811 full cell [15,S2] |
| --- | --- | --- |
| Mass loading of cathode (mg cm^-2^) | 30 | 30 |
| Active materials fraction (wt%) | 90 | 90 |
| Areal capacity of cathode (mAh cm^-2^) | 5.7 | 5.7 |
| Mass loading of SE layer (mg cm^-2^) | 3.5 | 3.5 |
| Mass loading of anode (mg cm^-2^) | 30.5 | 2.05 |
| Active materials fraction (wt%) | 55 [S1] | 85 |
| Areal capacity of anode (mAh cm^-2^) | 6.2 | 6.2 |
| Average voltage (V) | 3.7 | 3.5 |
| Energy density (Wh kg^-1^) | 327 | 553 |

The component and configurations of full-cells are described in the literature [S1, 15, S2]. Mass loadings are adjusted based on the theoretical capacities of LiNi_0.8_Co_0.1_Mn_0.1_O_2_(NCM811) (210 mAh g^-1^), graphite (372 mAh g^-1^) and Si (3579 mAh g^-1^) at an NP ratio of 1.1 [S3]. Mass loading of the SE layer corresponds to the thickness of ~20 μm [S3]. Energy densities are based on the mass loadings of the cathode, SE layer and anode, excluding the current collecting foils and wiring.

**Reference**

[S1] Sakuda, A., Kuratani, K., Yamamoto, M., Takahashi, M., Takeuchi, T. & H. Kobayashi, All-solid-state battery electrode sheets prepared by a slurry coating process, *J. Electrochem. Soc.* **164**, A2474–A2478 (2017).

[S2] Tan, D. H. S. *et al.* Carbon-free high-loading silicon anodes enabled by sulfide solid electrolytes. *Science* 373, 1494-1499 (2021).

[S3] Yim, C.-H., Mohamed S.E. Houache, M. S. E., Baranova, E. A. & Abu-Lebdeh, Y., Understanding key limiting factors for the development of all-solid-state-batteries. *Chem. Engineering J. Adv.* 13, 100436 (2023).

**Table S2.** Porosity of SiMg5.0 and SiMg7.5.

| Nanoporous Si fibers | BET specific surface area  (m^2^ g^-1^) | Total pore volume  (cm^3^ g^-1^) | Average pore size  (nm) |
| --- | --- | --- | --- |
| SiMg5.0 | 169 | 0.519 | 12.2 |
| SiMg7.5 | 109 | 0.266 | 9.7 |

**Table S3.** Initial charge and discharge performance of SiMg2.5, SiMg5.0, pulverized SiMg5.0 and nonporous Si.

| Nanoporous Si fibers | Initial charge/discharge capacity  (mAh g^-1^) | Initial Coulombic efficiency  (%) |
| --- | --- | --- |
| SiMg2.5 | 1124/530 | 47 |
| SiMg5.0 | 2411/1729 | 71 |
| Pulverized SiMg5.0 | 2017/853 | 42 |
| Nonporous Si | 3121/2487 | 80 |

**Eq. S1**

According to the literature [20, S4], we used Eq. S1 to estimate the Li ion diffusion coefficient, *D*_Li+,_ in SiMg5.0 from the Warburg impedance obtained by EIS measurement after the initial charge.

*D*_Li+_ = 1/2[(*V*_M_/*SFA*)(δ*E*/δ*x*)]^2^  (S1)

where *V*_M_ is mole volume of silicon, *S* is the surface area of the electrode, *F* is Faraday constant (96,486 C mol^-1^), *A* is the plot slope of imaginary resistance (*Z*_im_) vs. inverse square root of angular frequency ($1/\surd$(2π*f*)), which can be obtained from Warburg impedance, δ*E*/δ*x* is the slope of the galvanostatic charge-discharge curves.

The Li ion diffusion coefficient was found to be ~10^-12^ cm^2^ s^-1^, similar to the diffusion rate of Li within lithiated Si nanoparticles (~10^-12^ cm^2^ s^-1^) [S4] and lower than that of lithiated Si in micrometer-sized powder form (~10^-11^ cm^2^ s^-1^) [19].

**Reference**

[S4] Ding, N., Xu, J., Yao, Y.X. Wegner, G., Fang, X., Chen, C.H. & Lieberwirth, I. Determination of the diffusion coefficient of lithium ions in nano-Si. *Solid State Ionics*, **180**, 222-225 (2009).
